# Degradation of paper products due to volatile organic compounds

**DOI:** 10.1038/s41598-022-23898-z

**Published:** 2023-04-20

**Authors:** Izhar Alam, Chhaya Sharma

**Affiliations:** 1 Indian Institute of Technology Roorkee, Roorkee, India; 2 Department of Paper Technology, Indian Institute of Technology Roorkee, Saharanpur Campus, India

**Keywords:** Engineering, Materials science

## Abstract

Paper and packaging materials a lignocellulose-based natural biodegradable polymer that spontaneously releases acetic acid, aldehydes, alcohol, and ester-based volatile organic compounds (VOCs) upon ageing and these VOCs start degrading the paper products and decline their mechanical strength properties. The reactivity of the paper of unbleached wheat straw pulp towards acetic acid and hexanal, which has been proven to have more degrading effects on paper than other VOCs, was considered in this work. The papers were exposed to these volatile compounds for 90 days in an air-tight vessel under ambient environmental conditions. The results showed that hexanal was more destructive than acetic acid with regards to cellulose degradation and depletion in the mechanical strength properties. The paper properties like, tensile, tear and burst index, viscosity, pH and carbonyl group content was measured. The growth of the carbonyl group, evidence of the ageing effects in the paper, detected more in the paper exposed to acetic acid. However, the strength of paper properties declined more with hexanal. FE-SEM analysis of the sample showed the development of pores and damage of cellulose fibre upon ageing. Similarly, the damaging effects of VOCs on cellulose, hemicelluloses and lignin were confirmed by significantly reduced peak detection through FT-IR \analysis. The high crystallinity index of the paper products due to exposure to VOCs was detected by XRD analysis, which confirmed the degradation of the low molecular weight cellulose molecule. Thus, the results are strongly recommended that VOCs that generates due to natural or artificial ageing could be the leading cause of paper degradation.

## Introduction

The odour from old books, packaging material etc. might be because as its content, hundreds of recognized volatile and partially volatile organic compounds (VOCs), that are the result of off-gassing from the product^1,2^. The natures of VOCs are the result of decomposition pathways and the composition of the products, including chemical nature of the material and the chemical used to make the products. Previous work proposed that, oleic acid, a kind of extractives in the paper auto-oxidised to hydroperoxides which get further oxidised into a blend of VOCs such as octanal, nonanal, 2-decenal, 2-undecenal etc.^3^ and auto-oxidation of linoleic acid into hexanal and 2-octenal^4^. Experimental evidence suggested that different category of organic acids could be generated by the natural aging of all cellulose papers even after a few months of its manufacturing at ambient temperature^5^. As paper ages, it releases a combination of organic compounds, including acetic acid, formic acid and formaldehyde, which are corrosive in nature^6–8^ and the effects of acetic acid on degradation of paper has been demonstrated earlier^9^. Build up of acetic acid in a paper bundles may direct to more accelerated degradation and it was recently made known that emissions from one paper grade can have a significant negative impact on the humiliation of another paper grade^10^. Additionally, VOC emissions from mechanical pulp papers have been shown more degradation of cellulose papers in compared to bleach pulp paper^11^. Last from many years, there has been growing interest in the problem of interior pollutants in archives and libraries. Research has focused on assessment of generations from paper and books^8,12–22^ as a major source of VOCs and so far, slight identification on the possible hurtful effect of the VOCs on a paper product has been done. Significant quantity of acetic acid emission from archival cardboard storage boxes was identified by Dupont and Tetreault^23^ and evaluated the deterioration of paper due to this emission, as decrease in degree of polymerization of the cellulose in paper. Further, the exposures of paper to formic acid, 2-pentylfuran and NOx followed by high temperature treatment were performed earlier and observed with unfavourably influence the polymerization of cellulose, furthermore, acetic acid with modest effect on paper were identified with respect to, hexanal and furfural^24,25^. Despite these new findings, there are still gaps regarding the effects of VOCs on cellulosic materials and paper under different interior environmental conditions. There are many factors that affecting the degradation of cellulose within the paper, which mainly includes chemical process, such as acid hydrolysis, alkaline and oxidative degradation etc. The VOCs generation in a paper product are mainly due to the oxidative degradation process, oxidative degradation of cellulose is primarily induced by the presence of oxygen in the air of the environment. Oxidation reactions in a cellulose molecules introduces by side groups and probably get converted into aldehydes and ketones, which impart the cellulose molecules easier to hydrolyze. Oxidation reaction also induced formation of free radicals, which have capablity to break the chain of cellulose molecules, which may lead to an increase in the concentration of acids in the paper, as the reducing group, primary alcohol of the cellulose molecules can oxidised into carboxylic acid by photo-oxidation. Paper is biodegradable and comparatively stable material, but is subject to natural aging processes that cause cellulose degradation. Degradation of paper contributes to the formation of low molecular weight by-products/products viz., formic acid, acetic acid, etc., which can impart autocatalysis and leads to breakdown of cellulose chain^26^. Earlier study confirmed that organic acids with low-molecular weight are formed at the end of cellulose degradation, particularly the formic and acetic acids and furfural may also be generated due to the acid catalysed cellulose hydrolysis^8,27^. With regards to retard or minimize the degradation of paper, books and archival products, various technologies such as de-acidification and fibre strengthening have been developed^28,29^.

The aim of this work, unlike other’s finding, was to perform the exposure of VOCs such as acetic acid and hexanal under ambient condition (natural ageing), which are known for more damaging effects to the paper products in order to observe possible damage with regards to the mechanical properties of paper and the microscopic analysis of cellulose fibre to observe the morphological changes, that might occur due to VOCs.

## Experimental

### Materials

Unbleached pulp hand-sheets of wheat straw prepared in the laboratory was taken for the analysis. Hexanal of high purity were procured from Sigma-Aldrich and glacial acetic acid were from Fischer Scientific. In another work of VOCs generation from unbleached pulp/corrugated board, acid and aldehyde functionalized VOCs were identified among which, acetic acid and hexanal to be the most prominent^30,31^. Thus we were performed the degradation experiments with these two types of VOCs.

### Methods

#### Sample preparation

Unbleached pulp was prepared by cooking air dried and chopped wheat straw by kraft process in a digester (Rotary type) contains 4 stainless steel bomb with 100 gm oven dried weight material capacity (Universal Engineering Corporation, India, Model: UEC-2015). A known amount of straw as oven dry weight was taken and 18% active alkali (100 gpl solution) as Na_2_O was added into the bomb and certain amount of fresh water was also added to make 4:1 bath ratio (liquid to solid ratio). Subsequently, the bomb was kept in the digester, where water as a heating medium. Cooking of the material was performed at a temperature of about 165 °C for 30 min excluding the time required to reach targeted temperature, which was 60 min. After cooking, the pulps was washed thoroughly with hot and afterwards with normal water to take away the black liquor from the pulp until the pH of the filtrate reached to neutral. Before hand-sheets preparation, the pulp was refined to achieve 30 ± 2˚SR freeness level, tested in a Schopper-Reigler freeness tester (Lorentzen & Wettre, Stockholm, Sweden) in a PFI mill. Subsequently, the hand-sheets were preparation by disintegrating the pulp for about 5–10 min in a disintegrator and about 0.3% pulp slurry was ready to prepare with clean tape water. The hand sheet of about 250 ± 5 GSM was prepared by taking the known volume of the pulp slurry in a British sheet former following Tappi standard method, T 205 sp-02^32^.

#### Experiment for degradation

**Figure1:**
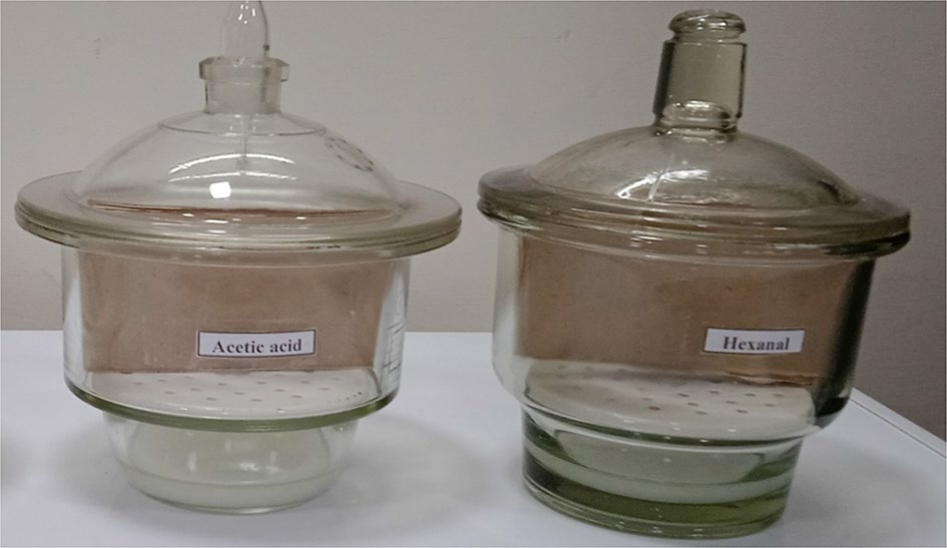
Paper exposed to VOCs in a desiccators.

The experiments were done in airtight desiccators, as a closed vessel with VOCs-water-salt a three constituent system were employed, following the earlier work^33^. The experimental setup was based on the water vapour equilibrium with mixture of saturated salt and VOCs, which creates equilibrium among the gas and liquid phase. 75% relative humidity (RH) was maintained by Sodium chloride at an ambient temperature^34^ and the VOCs solution was added to the saturated salt solution just prior to the sample was kept in the closed air tight vessel. To prepare VOCs-water-salt mixture, 50 ml VOCs solution (3 ml VOCs + 47 ml of water) were used to mix with 250 g of salt and placed in the desiccators. At the same time control samples, as a reference sample (RF) were placed in separate desiccators to expose with water-salt mixture only, which was denoted as denoted as RF and the paper exposed with acetic acid and hexanal are denoted as VAA and VH respectively. The hand-sheets were placed in the desiccators immediately over the surface of salt solution as shown in Fig. 1. After 30, 60 and 90 days exposure time completed, the samples were kept at ambient environmental conditions to remove the VOCs engrossed by the sample prior to the analysis.

#### Analysis of sample

##### pH determination

The pH of the sample was determined by hot extraction method according to Tappi standard T 435 om-02^35^.

##### Viscosity and degree of polymerization (DP)

Viscosity of the sample indicates the average degree of polymerization of a cellulose molecule. The degree of polymerization is a measure of the average length of a polymer in a solution, which was measured by the measurement of reflux time of cellulose solution which was dissolved in 0.5 M cupirethylenediamine (CED) solution in a capillary viscometer tube (Cannon–Fenske, model-100), according to Tappi standard, T 230 om-08 “Capillary viscometer method”^36^ and the values was calculated using arbitrary Eq. 1 and based on this value the intrinsic viscosity was obtained using a conversion table of T 206 om-55, using intrinsic viscosity, DP was calculated as below Eq. 2^37^.

1 $${\text{V }} = {\text{ C }} \times {\text{ t }} \times {\text{ d}}$$

2 $${\text{DP}}^{{0.{85}}} = { 1}.{1 } \times {\upeta }$$

where, V = Viscosity, mpa.s (cP) at 25 °C of cupirethylenediamine solution, C = Viscosity constant of capillary tube found by calibration, t = Average reflux time, s, d = Density of pulp solution, g/cm^3^ (1.052), η = intrinsic viscosity.

##### Physical Properties

The mechanical properties of handsheets viz., tear, burst and tensile breaking length were measured by following the Tappi standard method T 220 sp-01 “physical testing of pulp handsheets”^38^. Moreover, the hand-sheets were held in reserve for conditioning at temperature 27 ± 2 °C and a relative humidity of 65 ± 2% for about 24 h as per ISO 187:1990^39^ “Paper, board and pulps — Standard atmosphere for conditioning and testing procedure for monitoring the atmosphere and conditioning of samples” before the further analysis of physical properties.

##### Copper number (CN)/Carbonyl content

Copper number generally represents the reducing compounds in a paper^40^ and it is the number of gram metallic copper, can be defined as the number of grams of metallic copper, as Cu_2_O, consequent from the reduction of CuSO_4_ by 100 g of the sample.. These are primarily carbonyl functionalities in cellulose molecules arise from the C-1 end-group and carbonyl groups along C-2, C-3, and C-6 of the polymer. The C-1 end-group, lead to cellulose chain scission and the others carbons are indicative of cellulose oxidation, which converted into aldehydes and acidic compounds. Analysis was performed and followed according to the Indian Standard, IS: 6213-part VI^41^ and the carbonyl content was calculated using below Eqs. 3 and 4^40^.

3 $${\text{CN }} = \, \left( {{6}.{\text{36 x V x N}}} \right) \, /{\text{ W}}$$

4 $${\text{Content of Carbonyl group }}\left( {\mu \, {\text{mol}}/{\text{g}}} \right) \, = \, \left( {{\text{CN }}{-} \, 0.0{7}} \right) \, / \, 0.{6}$$

where, V (ml) = Volume of KMnO_4_ Solution required for titration, N = Normality of KMnO_4_ Solution, W (gram) = Weight of oven dried sample.

##### Image analysis

Morphological changes to the sample might occurred due to the degradation effects of VOCs was analysed using Field Emission Electron Microscope (TESCAN MIRA3; Brno, Czech Republic) techniques, that mechanism with electrons as an alternative source of light. Electrons are generated from tungsten filament by heating it to a temperature at about 2800 °C and After that the samples was scanned by these electrons and the images of samples were taken at different magnifications.

##### X-Ray Diffraction

X-ray diffraction analysis was performed for analysing the atomic and molecular structure of the material by quantifying the intensity of incident and scattered/reflected X-rays at particular angle of orientation of the rays right the way through the material. The diffraction patterns was generated using a X-ray diffractometer (Rigaku Ultima), instrument, the operating conditions was, 40 kV/40 mA, the range of angle of diffraction was set at θ = 5–80˚, at a scan rate of 4̊/minute. Crystalline index (CI) was obtained by calculating the difference between the lowest intensity (Iam) of the amorphous band and the peak of maximum intensity (Ic), divided by the peaks of maximum intensity (Ic) as presented in the Eq. 5.

5 $${\text{CI }} = \, \left[ {\left( {{\text{Ic }} - {\text{ Iam}}} \right)/{\text{ Ic}}} \right]{\text{ x 1}}00$$

##### Infrared spectroscopy

The information regarding specific functional group of the materials can be identified by this technique. Any irregularities or changes in the absorption band frequency may indicate the changes of chemical structure of the material. Fourier transform infrared spectroscopy (FTIR) of the sample was performed by attenuated total reflectance (ATR) method at ambient temperature. The spectra at 450 – 4000 cm^-1^ range as a result of 16 scans at a resolution of 4 cm^-1^ was corrected to recognize the functional groups.

## Result and discussion

Through exposing the paper to the volatile compounds at room temperature and relative humidity, the effects of individual volatile compounds were examined. The current research was done with slightly different approach as the past studies was performed for individual VOCs exposed at and before the hygrothermal treatment of paper^33,42,43^. This research observed that, exposure with acetic acid and hexanal without hygro-thermal treatment of the material, a considerable damage was observed after 90 days as presented in Tables 1,2 and 3, which was resulted in a decreased burst index by 19.2% in the sample RF, although more reduction by about 45.6% and 64.9% was detected with VAA and VH respectively. The force needed to break the strip of a paper, which are affected by several parameters like, individual fibre strength, length and the bonding among the fibres in the network, is tensile strength and the results depicted in tables are as index values. The tensile index was investigated to get reduced by 3.9% for RF, even though this reduction was observed to 12.8 and 25.8% for VAA and VH, correspondingly the increased tear index reduction by 53.8% was observed with VH, even though the reduction was 19.8 and 39.6% respectively, for RF and VAA. The exposure with VOCs significantly modified the other properties of paper with considerable decreased in DP, which indicates the cellulose degradation by, 6.8%, 10.7% and 16.0% in favour of RF, VAA and VH respectively. Previous work proposed that defeat of fiber strength is primarily due to the cellulose depolymerization caused by acid catalyzed hydrolysis^44^. Exposure with VOCs could cause oxidation of the cellulose, which accounts for the growth of carbonyl group in the molecules. After 90 days of getting old, the samples exposed with hexanal confirmed a few mild yellowing, even as there was no visible difference in the control sample were noticed, moreover the changes in the visual appearance, slight yellowing of Whatman No.1 qualitative filter even after 30 days of exposure with acetic acid was identified^9^. Even before ageing, carbonyl content of 1.9 µmol/g in the paper was investigated, this is most likely due to the oxidation took place during pulp generations with active alkali. Moreover the carbonyl content was increased to 2.2, 2.4 and 2.2 µmol/g in the sample, RF, VAA and VH respectively. A correlation between carbonyl content and the degradation of cellulosic pulps was obtained linear during the accelerated aging. The terminal reducing units of cellulose oxidised by molecular oxygen in an alkaline medium reach to the formation of aldonic acid, during the reaction, superoxide ions are is produced, whichis believed to be unable to extract a hydrogen atom from glucose, however, disproportionation can occur and the hydroperoxides are formed can lead to the formation of hydroxyl radicals^45^ the braking of the chain of cellulose molecules.

**Table 1:** mechanical properties of unbleached pulp sheets of controlled sample.

Parameters	RF			
	1st day	After 30 days	After 60 days	After 90 days
Grammage (g/m^2^)	249 ± 2.3	249.0 ± 2.5	247.8 ± 2.4	246.3 ± 2.8
% Reduction in GSM	–	0.0	0.5	1.1
Thickness (µm)	264 ± 18.7	263 ± 11.5	265 ± 30.8	264 ± 39.5
Bulk (cc/g)	1.06 ± 0.04	1.06 ± 0.08	1.07 ± 0.06	1.07 ± 0.05
Burst Index (kpa.m^2^/g)	5.7 ± 0.5	5.5 ± 0.4	5.1 ± 0.3	4.6 ± 0.2
Tensile Index(N.m/g)	71.3 ± 2.4	71.2 ± 3.7	70.8 ± 2.4	68.5 ± 1.83
Breaking Length (km)	7.3 ± 0.24	7.3 ± 0.38	7.2 ± 0.47	7.0 ± 0.19
Elongation (%)	4.5 ± 0.13	3.8 ± 0.20	2.9 ± 0.44	3.4 ± 0.09
Tear Index (mN.m^2^/g	9.1 ± 0.26	8.7 ± 1.42	7.4 ± 0.27	7.3 ± 1.44
Viscosity (cP)	11.9 ± 0.30	11.2 ± 0.25	11.0 ± 0.20	10.8 ± 0.15
Degree of Polymerization (No.)	1418 ± 42	1360 ± 55	1341 ± 58	1322 ± 40
Carbonyl content (µmol/g)	1.9 ± 0.08	1.9 ± 0.06	2.0 ± 0.30	2.2 ± 0.35
pH	7.8 ± 0.4	9.2 ± 0.1	9.6 ± 0.2	9.7 ± 0.1

**Table 2:** Mechanical properties of unbleached pulp sheets exposed to Acetic acid.

Parameters	VAA			
	1st day	After 30 days	After 60 days	After 90 days
Grammage (g/m^2^)	249 ± 2.3	256 ± 2.4	255 ± 2.1	254 ± 1.9
Enhanced GSM (%)	–	+ 2.9	+ 2.3	+ 1.8
Thickness (µm)	264 ± 18.7	265 ± 28.4	268 ± 34.9	257 ± 21.6
Bulk (cc/g)	1.06 ± 0.04	1.03 ± 0.02	1.05 ± 0.5	1.01 ± 0.03
Burst Index (kpa.m^2^/g)	5.7 ± 0.5	5.0 ± 0.8	3.5 ± 0.4	3.1 ± 0.6
Tensile Index(N.m/g)	71.3 ± 2.4	63.2 ± 2.63	62.2 ± 6.05	61.8 ± 3.17
Breaking Length (km)	7.3 ± 0.24	6.4 ± 0.27	6.4 ± 0.60	6.3 ± 0.32
Elongation (%)	4.5 ± 0.13	4.1 ± 0.35	3.2 ± 0.59	3.8 ± 0.20
Tear Index (mN.m^2^/g	9.1 ± 0.26	8.1 ± 1.15	7.8 ± 1.06	5.5 ± 0.36
Viscosity (cP)	11.9 ± 0.3	10.6 ± 0.34	10.5 ± 0.20	10.2 ± 0.30
Degree of Polymerization (No.)	1418 ± 42	1303 ± 68	1284 ± 66	1266 ± 72
Carbonyl content (µmol/g)	1.9 ± 0.08	2.1 ± 0.11	2.5 ± 0.50	2.4 ± 0.20
pH	7.8 ± 0.4	6.9 ± 0.4	5.3 ± 0.7	5.3 ± 0.7

**Table 3:** Mechanical properties of unbleached pulp sheets exposed to Hexanal.

Parameters	VH			
	1st day	After 30 days	After 60 days	After 90 days
Grammage (g/m^2^)	249 ± 2.3	257 ± 1.8	269 ± 2.1	294 ± 2.4
Enhanced GSM (%)	–	+ 3.0	+ 8.1	+ 18
Thickness (µm)	264 ± 18.7	267 ± 15.3	292 ± 8.4	310 ± 27.4
Bulk (cc/g)	1.06 ± 0.04	1.04 ± 0.03	1.08 ± 0.07	1.06 ± 0.05
Burst Index (kpa.m^2^/g)	5.7 ± 0.5	4.1 ± 0.3	3.4 ± 0.4	2.0 ± 0.7
Tensile Index(N.m/g)	71.3 ± 2.4	67.5 ± 1.05	55.4 ± 1.02	52.9 ± 4.24
Breaking Length (km)	7.3 ± 0.24	6.9 ± 0.11	5.7 ± 0.51	5.4 ± 0.43
Elongation (%)	4.5 ± 0.13	2.8 ± 0.21	2.9 ± 0.05	2.4 ± 0.05
Tear Index (mN.m^2^/g	9.1 ± 0.26	7.9 ± 0.06	7.0 ± 1.23	4.2 ± 0.05
Viscosity (cP)	11.9 ± 0.3	9.8 ± 0.15	9.7 ± 0.05	9.4 ± 0.1
Degree of polymerization (No.)	1418 ± 42	1228 ± 57	1209 ± 54	1191 ± 48
Carbonyl content (µmol/g)	1.9 ± 0.08	2.4 ± 0.22	2.4 ± 0.31	2.2 ± 0.31
pH	7.8 ± 0.4	6.4 ± 0.1	5.2 ± 0.2	4.9 ± 0.1

The breaking length of the samples was inconsistent with the other measurements, decreasing by 4.1% for the control sample, although upon exposure with VOCs, the decreased percentage was 13.8 and 26.0 for VAA and VH correspondingly. This reduced breaking length of the sample can explained by the fact that, the oxidants under acidic conditions oxidized, hydroxyl group at C-1, C-2 and C-3 of cellulose fibre to carbonyl and carboxyl group and, causes oxidative depolymerisation, resulting in loss of cellulose fibre strength^38^, thus besides oxidation, significant acid hydrolysis of the cellulose fibre took place due to exposure of paper products with the VOCs. Along with the depletion in the mechanical properties of paper, the basis weight of the paper was noticed to be decrease in the case of RF, even though the increased value of basis weight was observed when exposed with VOCS, this might be due to a little quantity of VOCs was seems to be absorbed by the paper during exposure. The weight of the sample was seemed to be increased by 2.9%, but further exposure lead to decreased in the basis weight with acetic acid, although the effect was observed to be enhanced, during throughout the exposure with hexanal as shown in tables. After 90 days of interpretation with VOCs, slight difference in the visual appearance, yellowish in colour to VAH samples were observed more than VAA sample, although no differences was noticed in RF sample.

**Figure 2:**
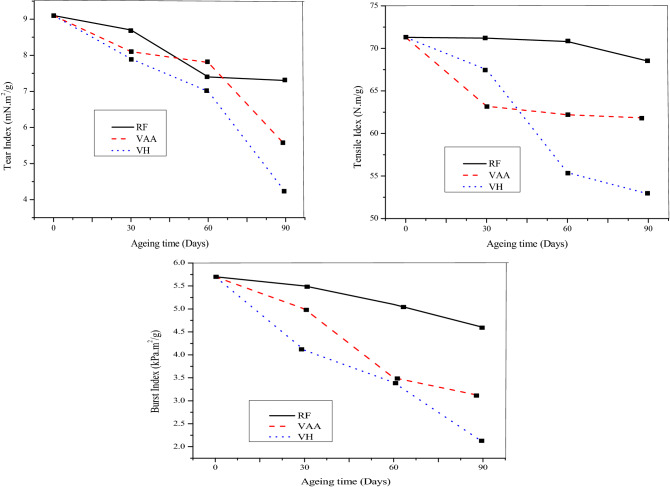
Effects of VOCs expose on mechanical properties of paper sheets.

**Figure 3:**
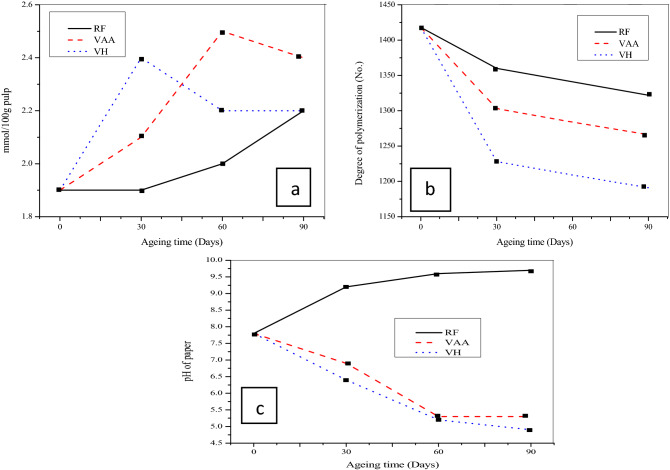
Effects on (**a**) carbonyl content, (**b**) DP, and (**c**) pH of paper due to expose in VOCs environments.

Thus more drops in the mechanical along with the other properties were observed with hexanal with respect to acetic acid and a clearer picture with regards to the damage in the paper could be seen from Figs.2 and 3. During the exposure, the pH of the samples seemed to be enhanced from the initial pH-7.8 to 9.7 for RF, although it was decreased from to 5.3 and 4.9 in the sample VAA and VH respectively. Thus more drop in pH was observed with hexanal and the pKa for hexanal is more than acetic acid, this could explain the elevated concentration of hydronium ions in the paper and lead to higher hydrolytic activity towards paper. Thus the study observed the depolymerization is predominantly due to acid hydrolysis, that caused by random cleavage of the cellulose fibre chain. Papers with a pH value above 4.5, tends to acid catalysed hydrolysis of cellulose in lesser extent, compare to at lower pH^44^. Acid hydrolysis causes chain cleavage of cellulose macromolecules to form fragments of carbohydrate molecules. These fragments get oxidized to carboxylic acids, which produce a cycle of oxidation and hydrolysis process, as a result which increased the acidity of the paper and cause an autocatalytic degradation process of the sample^46,47^.

**Figure 4:**
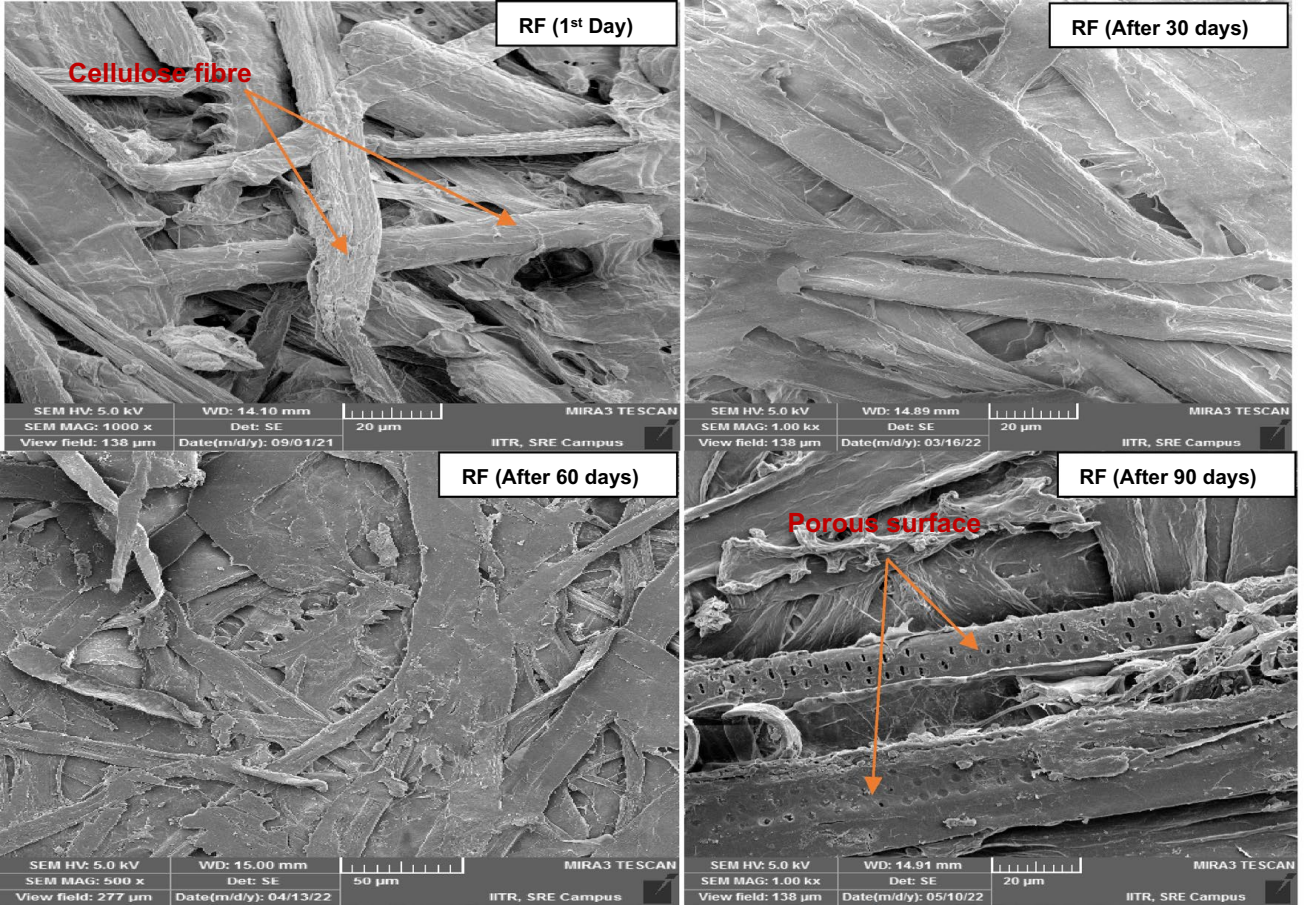
FE-SEM image analysis of the reference pulp sheet.

**Figure 5:**
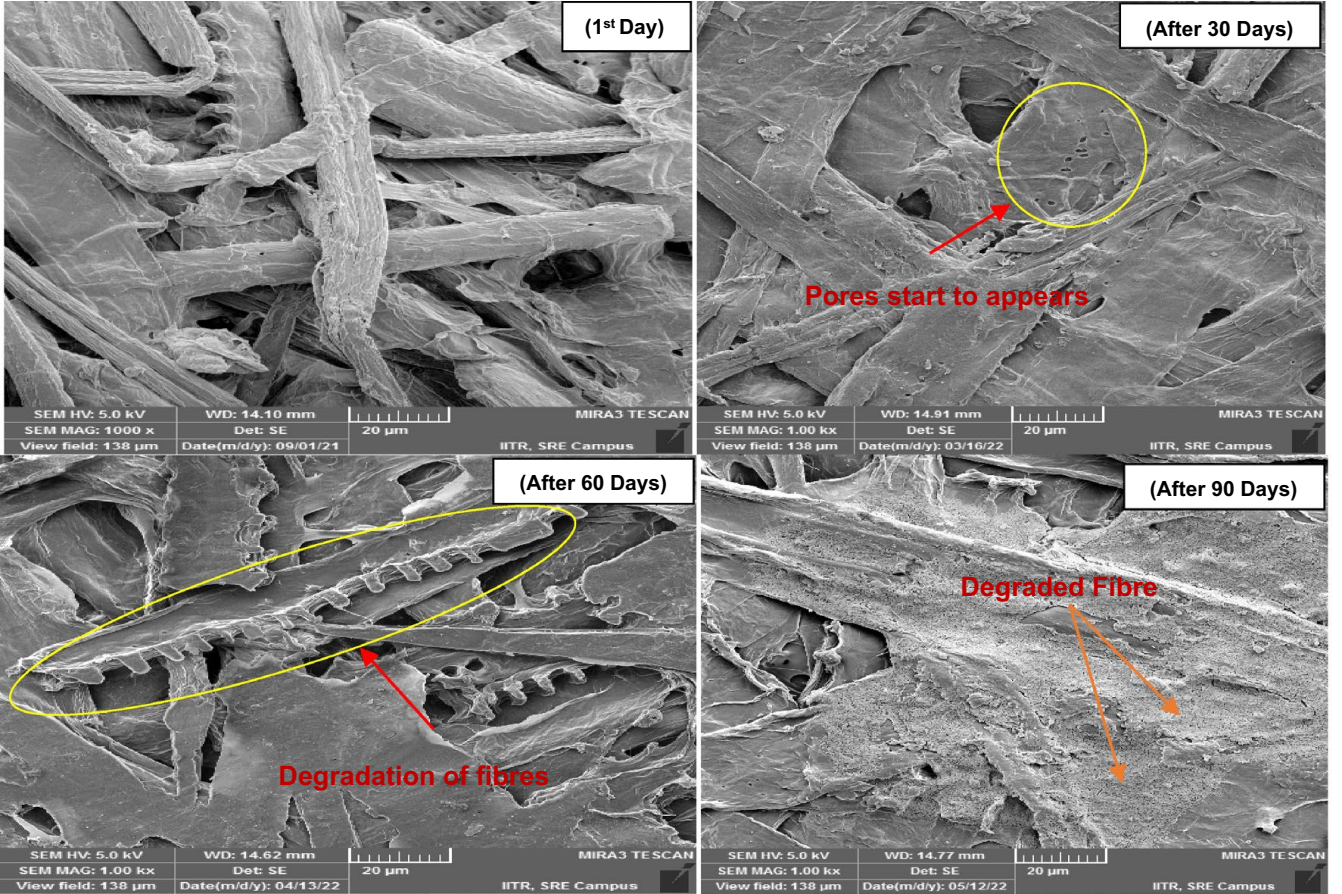
FE-SEM image analysis of the pulp sheet exposed to VAA.

**Figure 6:**
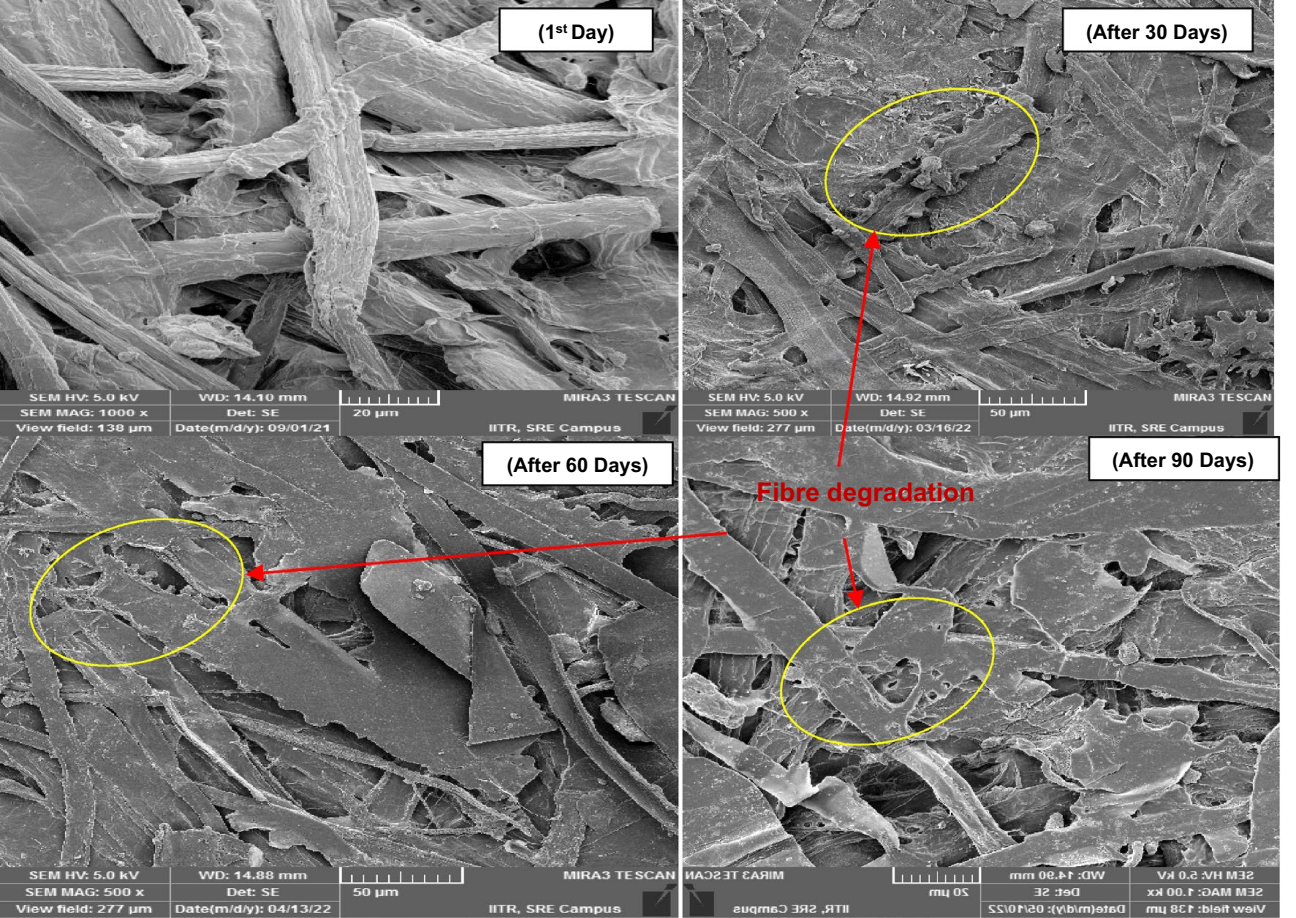
FE-SEM image analysis of the pulp sheet exposed to VH.

FE-SEM images were used to analyze the surface morphology of cellulose fibers upon aging of pulp sheets in a VOC environment. Figure 4 shows that cellulose fibers stored in a controlled environment (without VOCs) started to degrade after 60 days and developed a porous structure on the surface of the cellulose fibers after 90 days. As can be seen from Fig. 5, the pores were developed on the fiber surface even after 30 days of exposure and the degradation of cellulose fibers were observed after 60 days in VAA. Likewise in the sample VAA, the sample VH showed fibre damage even after 30 days of exposure as presented in Fig. 6.

**Figure 7:**
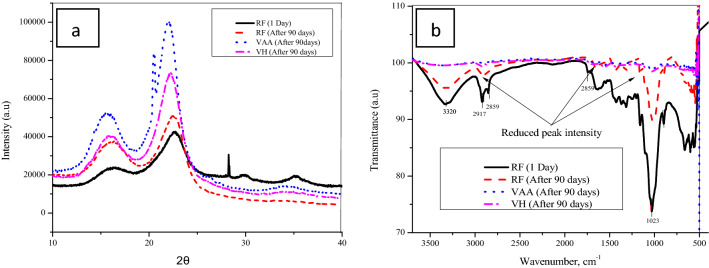
Characterization techniques (**a**) X-ray diffraction and (**b**) FTIR-ATR analysis of hand sheet exposed at VOCs environments.

XRD analysis was used to find out the changes in the crystallinity (CI) of cellulose fibre due to the VOCs. Figure 7a shows that, the presence of crystalline cellulose was indicated by a sharp and intense image at about 22.0° and the amorphous hemicellulose exhibited as a low reflection angle near to 15.0° approximately for all the samples. The RF sample initially represent a cristallinity index of 51.8%, after 90 days of ageing the CI was reduced to 51.2%, even though the CI of VAA and VH was observed to be increase to 66.6% and 60.3% respectively. The higher CI indicated higher cellulose content could be due to the degradation of some low molecular weight hemicellulose, lignin and other components, which was resulted into lower mechanical properties of the paper, as evident that hemicelluloses up to some extant enhanced the mechanical properties by cross-linking the long cellulose molecules.

FT-IR analysis was performed to understand the change or invariance of functional groups of cellulose fibers upon aging in VOCs environment. Figure 7b shows the FTIR spectra from 4000 to 500 cm^-1^ for the sample, initially a large peak band at 3320 cm^-1^ indicative of hydroxyl (OH) groups present in α-cellulose^48^ was observed for RF, but the peak area was found to be decreased after 90 days. Unlike RF, the peak intensity at 3320 cm^-1^ decreased at the end of exposure with both the VOCs, confirming that hydroxyl groups were converted to other functional groups that may serve as intermediates. The peaks recorded at 2917 and 2859 cm^-1^ confirms the presence of C–H stretch bands of cellulose components, which also appear to be diminished upon exposure. The band peak around 1734 cm^-1^ is due to the stretching of the C = O groups of hemicelluloses^49^. The peak at 1463 cm^-1^ corresponds to the C = H group of lignin content^50^. The corresponding peaks at 1314, 1245 and 1157 cm ^-1^ are the C–O–C bands of polysaccharides, C = H groups of lignin and C–O stretches of hydroxyl groups, respectively. The peak band recorded at ~ 1023 cm^-1^ is a C–OH stretch of lignin content^51^. A small band peak around ~ 719 cm^-1^ indicates a C–O–C stretch of monosaccharide content^52^. These peaks were significantly reduced in VAA and VH, confirming the degradation effects VOCs on all the pulp components including cellulose, hemicelluloses and lignin.

## Conclusion

Through this study, we were able to understand the effects of VOCs on paper degradation. The analysis was performed only for the individual two chemicals; using specific solvents and under specific conditions. The harmful impact of VOCs on paper humiliation depends on the types of VOCs involved. Volatile acids are generally the strongest, but aldehydes can accelerate decomposition to a similar degree. This study found that hexanal was more disruptive than acetic acid in reducing the mechanical properties of paper and the degree of polymerization of cellulose fibers. In addition to mechanical properties, effects on cellulose morphological properties such as porous structure and the degradation of the cellulose fibres are also noticed.

From the study, some important facts were pointed out that. the VOCs generated from paper based material could degrade it and preservation of the material can be achieve by minimizing the generation, which could be possible by developing a techniques for product manufacturing like, pre-treatment of raw material, different pulping process, different bleaching sequence can be evaluated, this may be the next level of research with regards to minimizing the generation of VOCs from the products. To better understand the qualitative assessment of the long-term effects of VOCs at room temperature, experiments should be conducted considering temperature and relative humidity. Future recommendations can be also derived that, removing VOCs can have a large positive effect on paper stability, potentially doubling life expectancy. .

## Data Availability

The database generated during this work may be obtainable from the corresponding author’s upon request.

## References

[1] Donetzhuber A, Johansson B, Johansson K, Lovgren M, Sarin E (1999). Analytical characterization of the gas phases in paper and board products. Nord. Pulp Pap. Res. J..

[2] Lattuati-Derieux A, Bonnassies-Termes S, Lave drine B (2004). Identification of volatile organic compounds emitted by a naturally aged book using solid-phase microextraction/gas chromatography/mass spectrometry. J. Chromatogr. A.

[3] Swoboda PAT, Lea CH (1965). The flavour volatiles of fats and fat-containing foods. II-A gas chromatographic investigation of volatile autoxidation products from sunflower oil. J. Sci. Food Agric..

[4] Matthews RF, Scanlan RA, Libbey LM (1971). Autoxidation products of 2,4,-decadienal. J. Americal Oil Chem. Soc..

[5] Shahani CJ, Harrison G (2002). Spontaneous formation of acids in the natural ageing of paper studied in conservation. Stud. Conversat..

[6] Buchbauer G, Jirovetz M, Wasicky M, Nikiforov A (1995). On the odour of old books. J. Pulp Pap. Sci..

[7] Lattuati-Derieux A, Bonnasies-Termes S, Lavédrine B (2004). Identification of volatile organic compounds emitted by a naturally aged book using solid-phase microextraction/gas chromatography/ mass spectrometry. J. Chromatogr. A..

[8] Strlic M, Cigic IK, Kolar J, De Bruin G, Pihlar B (2007). Non-destructive evaluation of historical paper based on pH estimation from VOC emissions. Sensors.

[9] Dupont AL, Tetreault J (2000). Cellulose degradation in an acetic acid environment. Stud. Conserv..

[10] Carter H, Bégin P, Grattan D (2000). Migration of volatile compounds through stacked sheets of paper during accelerated ageing. Part 1: Acid migration at 90 °C. Restaurator.

[11] Strlic M, Cigic IK, Mozir A, Thickett D, de Bruin G, Kolar J (2010). Test for compatibility with organic heritage materials–A proposed procedure. e-Preserv Sci..

[12] Ziegleder G (1998). Volatile and odorous compounds in unprinted paper board. Packag. Technol. Sci..

[13] Havermans, J.B.G.A., deFeber, M.A.P.C., Genuit, W.J.L., van Velzen, G.J. Emission of volatile organic compounds from paper objects affected with iron-gall ink corrosion. In* Preprints of the ICOM committee for conservation 12th trien-nial meeting*. **2**, 513–516 (1999).

[14] Pedersoli JL, Ligterink FJ, van Bomme lM (2011). Non-destructive determination of acetic acid and furfural in books by solid-phase micro-extraction (SPME) andgaschromatography-massspectrometry(GC/MS). Restaurator.

[15] Doering T, Fischer P, Banik G, Binder U, Liers J (2001). An approach to evaluate the condition of paper by a non-destructive analytical method. Adv. Print Sci. Technol..

[16] Shahani CJ, Harrison G, Daniels V, Donnithorne A, Smith P (2002). Spontaneous formation of acids in the natural aging of paper. Works of art on paper books, documents and photographs: Techniques and conservation.

[17] Lattuati-Derieux A, Bonnassies-Termes S, Lavédrine B (2006). Characterisation of compounds emitted during natural and artificial ageing of a book. Use of headspace-solid-phase micro-extraction/gas chromatography/mass spectrometry. J. Cult. Herit..

[18] Strlic, M., Cigic, K.I, Kolar, J., de Bruin, G., Steemers, T. The Paper VOC project: measurement and simulation of VOC emissions from paper. In *Past-present - prediction: about simulation techniques, dosimeters, sensors, in conservation research and application, COST strategic work shop*. Ohrid, FYR of Macedonia (2007).

[19] Dupont AL, Egasse C, Morin A, Vasseur F (2007). Comprehensive characterisation of cellulose and lignocelluloses degradation products in aged papers: Capillary zone electrophoresis of low molar mass organic acids, carbohydrates, and aromatic lignin derivatives. Carbohydr. Polym..

[20] Gaspar EM, Santana JC, Lopes JF, Diniz MB (2010). Volatile organic compounds in paper–an approach for identification of markers in aged books. Anal. Bioanal. Chem..

[21] Clark AJ, Calvillo JL, Roosa MS, Green DB, Ganske JA (2011). Degradation product emission from historic and modern books by head space SPME/GC-MS: Evaluation of lipid oxidation and cellulose hydrolysis. Anal. Bioanal. Chem..

[22] Jablonský M, Katuscák S, Holúbková S, Hrobonová K, Lehotay J (2011). The effect of acetic and formic acid formation during accelerated ageing on embrittlement of news printpaper. Restaurator..

[23] Dupont AL, Tétreault J (2000). Study of cellulose degradation in acetic acid environments. Stud. Conserv..

[24] Menart E, De Bruin G, Strlic M (2011). Dose-response function for historic paper. Polym. Degrad. Stab..

[25] Strlic M, Cigic KI, Mozir A, de Bruin G, Kolar J, Cassar M (2011). The effect of volatile organic compounds and hypoxia on paperd egradation. Polym. Degrad. Stab..

[26] Baranski A, Dziembaj R, Konieczna-Molenda A, Lagan JM, Walas S (2004). On the applicability of arrhenius equation to accelerated ageing tests. The case of alumimpregnated cellulose. Pol. J. Chem. Technol..

[27] Nevell TP, Zeronian SH (1985). Cellulose chemistry and its applications. Jour. Poly. Sci..

[28] Ramin M, Andres H, Blüher A, Reist M, Wälchli M (2009). Paper de-acidification – A comparative study. J. Paper Conserv..

[29] Proniewicz LM, Paluszkiewicz C, Wesenucha-Birczynska A, Majcherczyk H, Baranski A, Konieczna A (2001). FT-IR and FT-raman study of hydrothermally degradated cellulose. J. Molec. Struc..

[30] Hamalainen H, Ekman K, Lassila P, Jakara J (2005). On the management of odour in chemical pulps: Formation and removal Hexanal. Appita Annu. Conf..

[31] Jablonsky M, Katuscak S, Holubkova S, Hrobonova K, Lehotay J (2011). The effect of acetic acid and formic acid formation during accelerated ageing on embrittlement of news print paper. Restaurator..

[32] T 205sp-02 “Forming handsheets for physical test of pulp” 1-13 (2006).

[33] Tétreault J, Dupont A-L, Bégin P, Paris S (2013). The impact of volatile compounds released by paper on cellulose degradation in ambient hygrothermal conditions. Polym. Degrad. Stab..

[34] Greenspan L (1977). Humidity fixed points of binary saturated aqueous solutions. J. Res. Bur. Stand Sect A Phys. Chem..

[35] T 435om-02 “ Hydrogen ion concentration of paper extract (Hot extraction method)” 1–7 (2006).

[36] T 230 om-08 “ Viscosity of pulp (capillary viscometer method)” WI-120809.04, 1–13 (2006).

[37] Setu MNI, Mia MY, Lubna NJ, Chowdhury AA (2014). Preparation of microcrystalline cellulose from cotton and its evaluation as direct compressible excipient in the formulation of Naproxen tablets. Dhaka Univ. J. Pharm. Sci..

[38] T 220 sp-01 “physical testing of pulp handsheets” 1–6, (Revised-2001)

[39] ISO 187:1990 “Paper, board and pulps—Standard atmosphere for conditioning and testing procedure for monitoring the atmosphere and conditioning of samples” (1990–12).

[40] Röhrling J, Potthast A, Rosenau T, Lange T, Borgards A, Sixta H (2002). A novel method for the determination of carbonyl groups in cellulosics by fluorescence labelling 2. Validation and applications. Biomacromolecules..

[41] IS: 6213-part VI “ Determination of copper number” (1971).

[42] Menart E, De Bruin G, Strlc M (2011). Dose-response function for historic paper. Polym. Degrad. Stab..

[43] Strlic M, Cigic KI, Mozir A, de Bruin G, Kolar J, Cassar M (2011). The effect of volatile organic compounds and hypoxia on paper degradation. Polym. Degrad. Stab..

[44] Zou X, Gurnagul N, Vesaka T, Bouchard J (1994). Accelerated ageing of paper of pure cellulose: Mechanism of cellulose degradation and paper embrittlement. Polym. Degrad. Stab..

[45] Malesic, J., Kolar, J., and Strlič, M. Effect of pH and carbonyls on the degradation of alkaline paper - Factors affecting ageing of alkaline paper. *Restaurator*. **23**, 145–153 ((1994).

[46] Lai Y-Z, Hon N-S, Shiraishi N (2001). Chemical degradation. Wood and cellulosic chemistry.

[47] Shahani, C. J. Can accelerated aging foretell the permanence of paper. In *ASTM Workshop on the Effects of Aging on Printing and Writing Papers*, West Conshohocken, PA: ASTM I, 120–139 (1994).

[48] Shahani, C. J., Harrison, G.: Spontaneous formation of acids in the natural aging of paper. In: Preprints to the IIC Congress, Baltimore, 2002, Works of Art on Paper, Books, Documents and Photographs: Techniques and Conservation, V. Daniels, V. A. Donnithorne, P. Smith (eds), London: IIC, 189–192 (2002).

[49] Bessa W, Trache D, Derradji M, Tarchoun AF (2021). Morphological, thermal and mechanical properties of benzoxazine resin reinforced with alkali treated alfa fibers. Ind. Crops Prod..

[50] Singh JK, Rout AK, Kumari K (2021). A review on Borassus flabellifer lignocellulose fiber reinforced polymer composites. Carbohydr. Polym..

[51] Ganapathy T, Sathiskumar R, Senthamaraikannan P, Saravanakumar SS, Khan A (2019). Characterization of raw and alkali treated new natural cellulosic fibres extracted from the aerial roots of banyan tree. Int. J. Biol. Macromol..

[52] Sabarinathan P, Annamalai VE, Rajkumar K, Vishal K, Dhinakaran V (2022). Synthesis and characterization of randomly oriented silane-grafted novel bio-cellulosic fish tail palm fiber– reinforced vinyl ester composite. Biomass Convers Biorefinery..

